# Impact of carboplatin desensitization therapy on progression-free survival in gynecologic cancers

**DOI:** 10.1016/j.gore.2025.102017

**Published:** 2025-12-29

**Authors:** Nikita Bastin, Halle Petrie, Marc Robinson, Amir Javid, Robin Lane, Taryn Boucher, Alaina J. Brown, Lauren S. Prescott, Elizabeth Phillips, Ronald D. Alvarez, Marta Crispens, Cosby A. Stone

**Affiliations:** a Vanderbilt University School of Medicine, Nashville, TN, USA; b Vanderbilt University Medical Center, Nashville, TN, USA; c Vanderbilt Department of Obstetrics and Gynecology, TN, USA; d Vanderbilt Department of Allergy, Pulmonary and Critical Care Medicine, USA

**Keywords:** Carboplatin allergy, Gynecologic cancer, Survival, Desensitization

## Abstract

**Objectives:**

To compare survival between gynecologic cancer patients with carboplatin hypersensitivity thereafter managed with carboplatin desensitization, cisplatin replacement, and alternative non-platinum therapy.

**Methods:**

A retrospective review of patients who experienced a hypersensitivity reaction to carboplatin and were treated at the same comprehensive cancer center between 2010 and 2024 was completed. The primary analysis compared progression-free survival between carboplatin desensitization, cisplatin substitution, and alternative non-platinum therapy patients. Secondary analysis compared survival between platinum therapy and non-platinum alternative therapy patients. Descriptive analysis was further performed to delineate the clinical phenotypes of patients with carboplatin hypersensitivity.

**Results:**

There were 47 gynecologic cancer patients with carboplatin hypersensitivity, and 38 patients with known progression-free survival. Progression-free survival was significantly longer in desensitized patients (31.1 months) vs. cisplatin patients (22.0 months, p = 0.045). There were no significant differences in progression-free survival between desensitized (31.3 months) and alternative therapy patients (36.0 months), as well as platinum (31.1 months) and non-platinum-based alternative therapy patients (36.0 months). A majority of our cohort was observed to have recurrent disease at the time of hypersensitivity (70.2 %), a carboplatin-free interval of 12 months or more (57.4 %), history of adverse reactions to other drugs (76.6 %), advanced stage disease (78.8 %), and serous histology (70.2 %).

**Conclusions:**

Desensitized patients demonstrated increased progression-free survival as compared to cisplatin patients. This progression-free survival advantage may be due to the increased toxicity profiles noted for cisplatin and comparatively better tolerability of carboplatin desensitization. Progression-free survival was similar between carboplatin-desensitized and alternative therapy patients, as well as platinum-based and alternative therapy patients.

## Highlights

• This is the first study comparing treatment strategies after carboplatin hypersensitivity in gynecologic cancer.
• Gynecologic cancer patients had longer progression-free survival with carboplatin desensitization than cisplatin.
• Carboplatin desensitization may improve progression-free survival due to its comparatively better tolerability profile.
• Progression-free survival was similar between platinum-based therapy and non-platinum alternative therapy.

## 1 Introduction

Carboplatin is an integral part of the treatment of several gynecologic cancers, including endometrial and ovarian cancers of various histologies (Caruso et al., 2025, Matei et al., 2019). However, carboplatin hypersensitivity reactions, including anaphylaxis, have been noted to occur in 7–15 % of gynecologic oncology patients (Navo et al., 2006, Tai et al., 2017, Paksoy et al., 2022). Various risk factors for developing a carboplatin hypersensitivity reaction have been elucidated, including higher cumulative number of carboplatin cycles, history of prior allergies, BRCA1/2 mutation status, advanced stage or recurrent disease, and longer platinum-free interval (Tai et al., 2017, Jang et al., 2025).

These reactions pose a significant challenge to patients as discontinuation or switching to non-platinum regimens is potentially associated with inferior oncologic outcomes (Narui et al., 2019). In fact, prior studies have shown patients with carboplatin desensitization have similar overall survival to patients without carboplatin hypersensitivity (Paksoy et al., 2022, Zwimpfer et al., 2024, Altwerger et al., 2018, Li and Yin, 2024). While switching to a non-cross reactive platinum-based therapy has not been directly compared to carboplatin desensitization, it has been noted to lead to improved overall survival as compared to switching to a non-platinum based therapy, with a difference of about 13 months (Altwerger et al., 2018). Even when cisplatin is an available alternative from an allergy standpoint, the nonhematologic side effects of cisplatin may still lead to carboplatin desensitization being a favored approach (Lorusso et al., 2014, Ho et al., 2016, Go and Adjei, 1999, Lokich and Anderson, 1998).

There are currently no studies that have directly compared survival between gynecologic cancer patients managed with carboplatin desensitization, cisplatin replacement, and alternative non-platinum therapy. Several retrospective studies have provided indirect comparisons of these treatment strategies. This study aims to address a critical gap in the medical literature by directly comparing the oncologic outcomes among gynecologic cancer patients who experienced a hypersensitivity reaction to carboplatin and received one of three possible subsequent therapeutic modifications, including carboplatin desensitization, cisplatin substitution, or an alternative therapy. The secondary objective was to describe the clinical phenotypes of patients with carboplatin allergy.

## 2 Methods

### 2.1 Study sample

This study was an Institutional Review Board (IRB) approved retrospective review of patients with a gynecologic malignancy who experienced a hypersensitivity reaction to carboplatin and were treated at the same comprehensive cancer center between 2010 and 2024. Data were collected using the electronic medical record. This was a joint study conducted by the 10.13039/100013017Vanderbilt University Medical Center (VUMC) Department of Allergy, Pulmonary and Critical Care Medicine and the Division of Gynecologic Oncology.

Patient records were reviewed to ensure that all study eligibility criteria were met prior to initiating study enrollment and collecting additional data. Patients who were identified for study enrollment were those with gynecologic cancers who experienced carboplatin allergy as a part of their cancer treatment course. Inclusion criteria included women ≥ 18 years treated for gynecologic malignancies who received the majority of their care at Vanderbilt between 2010 and 2024 and whose subsequent treatment following an immediate hypersensitivity reaction to carboplatin was documented. Exclusion criteria included an inability to obtain information regarding treatment.

### 2.2 Desensitization Procedure

Desensitization became available as a routinely available treatment strategy at VUMC starting at the beginning of 2022. When chosen, desensitization was selected after risk-assessment and shared decision-making between the oncologist, allergist, and patient. Most desensitization procedures were performed in an inpatient setting, with a more recent implementation in 2024 that allows patients with well-tolerated desensitizations to receive them in the outpatient setting.

Desensitization to carboplatin was completed according to a 13-step pharmacy desensitization protocol using 3 bags at 1:100, 1:10, and 1:1 dilutions compared to a typical preparation. Breakthrough symptoms were promptly treated and per protocol would result in temporary cessation of treatment with resumption either at the same step or previous steps, depending on severity, once the symptoms had been adequately managed.

The standard desensitization premedication regimen was as follows: premedication with cetirizine 10 mg or fexofenadine 180 mg twice daily, famotidine 20 mg twice daily and montelukast 10 mg daily for the two days before, day of, and two days after desensitization, along with typical chemotherapy premedication such as dexamethasone.

### 2.3 Data Collection and analysis

Data were collected from the electronic medical record by study personnel trained in data extraction. Clinical data recorded included age, race, ethnicity, attending physician, zip code, insurance status, medical history, surgical history, and allergy history. Information regarding the patients’ initial reaction to chemotherapy as well as their desensitization regimens was further recorded. Laboratory history and medication history in the year prior to and following reactions were noted. Pathologic information about patients’ cancers was recorded, including histology of cancer, stage of cancer (I, II, III, IV), and presence of genetic testing. Treatment data, including receipt of interval debulking as well as optimal cytoreduction, were recorded. Historical features, such as a history of reactions to other medications were noted. Progression-free survival was recorded; it was defined as the time from diagnosis to disease progression following therapy.

Study data were collected and managed using REDCap electronic data capture tools housed within Vanderbilt University Medical Center.

Descriptive statistics were calculated. All analyses were performed using REDCap and R in August 2025. Treatment groups were controlled for potential confounding factors, including age, BRCA mutation status and recurrent disease status. For the primary analysis of progression free survival, subsequent treatments were classified in the following three categories: 1. carboplatin desensitization versus 2. switch to cisplatin versus 3. use of an alternative chemotherapy regimen. For the secondary analysis of progression free survival, subsequent treatments were classified into two categories 1. Continued use of a platinum drug (either by desensitization or use of a tolerated alternative), versus 2. Use of an alternative chemotherapy regimen. We used Cox-proportional hazard modeling to adjust for age, BRCA mutation status, and recurrent disease.

In accordance with the journal’s guidelines, we will provide our data for independent analysis by a selected team by the Editorial Team for the purposes of additional data analysis or for the reproducibility of this study in other centers if such is requested.

## 3 Results

### 3.1 Demographics

While 58 patients were identified to have immediate hypersensitivity/allergy to carboplatin, only 47 patients met our inclusion and exclusion criteria and therefore were included in our analysis. The patient cohort included 47 patients diagnosed with gynecological cancer and treated with carboplatin during the study period, who had experienced an immediate carboplatin hypersensitivity reaction. 39 (83.0 %) patients had ovarian cancer, 7 (14.9 %) patients had endometrial cancer, and 1 (2.1 %) patient had cervical cancer. Most patients had advanced stage disease with N = 21 (44.7 %) with stage III and N = 16 (34.1 %) with stage IV disease. Most patients had cancer that was high grade (70.2 %) and of serous histology (70.2 %). Most patients also had genetic testing (87.2 %). The most common mutations noted in the cohort were BRCA1 (27.6 %) and TP53 (44.7 %). All patients received chemotherapy (100 %) and most patients had cytoreductive surgery (97.9 %). Patient demographics and treatments received are detailed in Table 1.

**Table 1:** Demographics.

**Characteristics**	**Carboplatin desensitization (17)**	**Cisplatin substitution (8)**	**Alternative therapy (22)**	**Total**
Age: Median (IQR)	60.0 (53.0, 67.0)	59.0 (48.0, 65.5)	64.0 (62.0, 75.0)	63.0 (57.0, 70.0)
Cancer Type: n (%)				
*Ovarian*	14 (82)	7 (88)	17 (81)	39 (83.0)
*Endometrial*	3 (18)	1 (13)	3 (14)	7 (14.9)
*Cervical*	0 (0)	0 (0)	1 (5)	1 (2.1)
Stage: n (%)				
*I*	2 (11.8)	0 (0)	3 (13.6)	5 (10.6)
*II*	1 (5.9)	1 (12.5)	3 (13.6)	5 (10.6)
*III*	7 (41.2)	4 (50.0)	10 (45.5)	21 (44.7)
*IV*	7 (41.2)	3 (37.5)	6 (27.3)	16 (34.1)
Grade: n (%)				
*High*	10 (58.8)	4 (50.0)	19 (86.4)	33 (70.2)
*Low*	1 (5.9)	1 (12.5)	1 (4.6)	3 (6.4)
*Other/Unknown*	6 (35.3)	3 (37.5)	2 (9.1)	11 (23.4)
Histology: n (%)				
*Serous*	12 (70.6)	4 (50.0)	11 (50.0)	33 (70.2)
*Clear Cell*	2 (11.8)	1 (12.5)	1 (4.6)	4 (8.5)
*Endometrioid*	1 (5.9)	1 (12.5)	2 (9.1)	5 (10.6)
*Carcinosarcoma*	2 (11.8)	0 (0)	1 (4.6)	3 (6.4)
*Other*	2 (11.8)	1 (12.5)	1 (4.6)	2 (4.3)
Genetic Testing: n (%)	12 (80.0)	7 (88.0)	18 (95.0)	37 (88.0)
Genetics: n (%)				
*Germline*				
*BRCA1*	7 (41.2)	1 (12.5)	5 (22.7)	13 (27.6)
*BRCA2*	1 (5.9)	0 (0)	2 (9.1)	3 (6.4)
*TP53*	10 (58.8)	2 (25.0)	9 (40.9)	21 (44.7)
*PTEN*	0 (0)	2 (25.0)	4 (18.2)	6 (12.8)
*Somatic*				
*PIK3CA*	3 (17.6)	1 (12.5)	1 (4.6)	5 (10.6)
*ARID1A*	3 (17.6)	0 (0)	3 (13.6)	6 (12.8)
Surgery: n (%)	17 (100.0)	7 (87.5)	22 (100.0)	46 (97.9)
Chemotherapy: n (%)	17 (100.0)	8 (100.0)	22 (100.0)	47 (100)
Radiation Therapy: n (%)	3 (17.6)	3 (37.5)	5 (22.7)	11 (23.4)
Cycle Number: Median (IQR)	7.0 (5.0, 9.0)	7.5 (4.0, 14.5)	8.5 (7.0, 12.0)	8.0 (6.0, 10.0)
BRCA mutation: n (%)	7 (41.2)	1 (13)	5 (23)	13 (27.6)
Recurrent disease at time of reaction: n (%)	11 (65)	4 (50)	18 (82)	33 (70.2)
Carboplatin free interval for 12 + months: n (%)	8 (47)	4 (50)	15 (68)	27 (57.4)
History of Adverse Reactions with other Drugs: n (%)	14 (82)	5 (63)	17 (77)	36 (76.6)

Among 38 patients whose progression free survival data were known, we observed the following proportions of subsequent treatments strategies: 12 (31.6 %) had received carboplatin desensitization, 5 (13.2 %) received carboplatin, and 21 (55.3 %) had received a non-platinum alternative regimen.

The median cycle number at the time of carboplatin hypersensitivity reaction was 8. A majority of our cohort was noted to have recurrent disease (70.2 %), carboplatin free interval for 12 + months (57.4 %), and a history of adverse reactions with other drugs (76.6 %). BRCA mutations were noted in 27.7 % of our cohort.

### 3.2 Treatment Timing and Description

Across all three cohorts, patients demonstrated long and heterogenous treatment courses. Their overall cancer treatment timelines and treatment courses following carboplatin allergy are delineated in Table 2. For alternative treatment patients, the rationale for treatment change if described in the electronic medical record is provided.

**Table 2:** Treatment Timings and Description.

Carboplatin Desensitization		
**Patient Number**	**Time Course of All Treatments**	**Treatments Following Carboplatin Allergy**
1	2021–2023	Carboplatin/Paclitaxel
2	2022–2023	Carboplatin/Paclitaxel, Letrozole
3	2019–2023	Carboplatin/Docetaxel, Pembrolizumab/Lenvatinib, Bevacizumab, Doxorubicin, Mirvetuximab soravtansine, MICA/MICB antibody
4	2015–2025	Carboplatin, Bevacizumab/Paclitaxel, Gemcitabine, Bevacizumab/Cyclophosphamide/Pembrolizumab
5	2020–2022	Carboplatin/Paclitaxel
6	2021–2025	Paclitaxel/Carboplatin/Dostarlimab
7	2022–2024	Carboplatin/Paclitaxel, Gemcitabine
8	2021–2024	Carboplatin/Paclitaxel/Pembrolizumab
9	2008–2018	Carboplatin, Radiation, Olaparib, Rucaparib, Doxorubicin, Paclitaxel/Bevacizumab, Olaparib
10	2020–2024	Carboplatin/Doxorubicin/Bevacizumab, Bevacizumab, Mirvetuximab Soravtansine
11	2019–2023	Carboplatin/Paclitaxel/Bevacizumab, Carboplatin/Doxorubicin
12	2020–2025	Carboplatin/Paclitaxel, Olaparib, Carboplatin/Doxorubicin
13	2019–2023	Carboplatin/Docetaxel
14	2022–2025	Carboplatin/Paclitaxel, Olaparib, Carboplatin/Doxorubicin/Bevacizumab
15	2020–2022	Carboplatin/Paclitaxel, Pembrolizumab/Lenvatinib, Pembrolizumab, Paclitaxel
16	2013–2023	Carboplatin/Paclitaxel
17	2023–2025	Carboplatin/Doxorubicin

b) Cisplatin Substitution		
**Patient Number**	**Time Course of All Treatments**	**Treatments Following Carboplatin Allergy**
1	2018	Cisplatin
2	2013–2025	Cisplatin/Doxorubicin, Bevacizumab/Cyclophosphamide, Zoledronic Acid, Gemcitabine, Letrozole/Everolimus
3	2004–2025	Cisplatin, Doxorubicin, Gemcitabine, Topotecan
4	2014	Cisplatin, Paclitaxel
5	2021–2025	Carboplatin/Paclitaxel, Pembrolizumab/Lenvatinib, Olaparib
6	2017–2018	Cisplatin/Paclitaxel, Olaparib
7	2014–2017	Cisplatin/ Docetaxel, Docetaxel
8	2017–2025	Cisplatin/Gemcitabine/Bevacizumab, Bevacizumab

(c) Alternative Therapy			
**Patient Number**	**Time Course of All Treatments**	**Treatments Following Carboplatin Allergy**	**Reasoning for Treatment Change**
1	2003–2018	Niraparib	Niraparib thought to be better option
2	2010–2017	Taxotere/Gemcitabine, single agent Taxotere, radiation	Taxotere thought to be better option
3	2012–2017	Femara, Aromasin, Topotecan, Abraxane, Letrozole	Allergic reaction was final Carboplatin cycle
4	2015–2020	Cisplatin, Taxol, Neulasta	Several Cisplatin cycles held due to kidney dysfunction
5	2018–2022	Gemcitabine, Bevacizumab	Rationale not described, care partially received at another institution
6	1999–2021	Taxol, Navelbine	Rationale not described, care partially received at another institution
7	2017–2022	Doxorubicin, Cisplatin/Gemcitabine, Olaparib, Rebastinic/Taxol, Nivolumab, Bevacizumab/Paclitaxel, Bevacizumab/Topotecan	Transitioned to consolidation taxol after carboplatin allergy
8	2020–2021	Rucaparib, radiation	PARP inhibitor thought to be better option
9	2013–2025	Lipodox, Tamoxifen, Olaparib, Surgery, Docetaxel, Cisplatin/gemcitabine, Doxorubicin, Topotecan, Cisplatin, Desensitized carboplatin, Bevacizumab/paclitaxel, Mirvetuximab, Cyclophosphamide, Etoposide	Rationale not described
10	2016–2020	Niraparib	Transitioned to maintenance therapy
11	2022–2024	Bevacizumab, Pembrolizumab, Doxorubicin	Discontinued platinum agents due to progression
12	2018–2018	No therapy	Died shortly after reaction
13	2015–2016	Radiation	Rationale not described
14	2019–2023	Doxorubicin/Bevacizumab, Pembrolizumab/Cytoxan/Bevacizumab, Batiraxcept/Paclitaxel, Topotecan/Bevacizumab, Gemcitabine	Rationale not described, care partially received at another institution
15	2021–2022	No therapy	Rationale not described
16	2020–2023	Doxorubicin	Rationale not described
17	2022–2024	Taxol/Bevacizumab	Care partially received at another institution, no option for desensitization there
18	2015–2022	Single-agent Paclitaxel, Everolimus/Letrozole, Pembrolizumab/Lenvatinib	Minimal disease after carboplatin reaction, transitioned
19	2015–2025	Cisplatin, Single-agent Paclitaxel, Cisplatin/Paclitaxel, Docetaxel, Olaparib, Desensitized Carboplatin/Doxorubicin, Cyclophosphamide, Gemcitabine, Pembrolizumab	Allergy to both Carboplatin and Cisplatin, transitioned to single agent paclitaxel
20	2019–2020	Single-agent Paclitaxel, Pembrolizumab, Everolimus/Letrozole, Radiation	Transitioned to palliative chemotherapy
21	2016–2022	Single-agent Paclitaxel, Cyclophosphamide/ Bevacizumab, Taxotere	Rationale not described, care partially received at another institution

Patients receiving carboplatin desensitization underwent desensitization following 2022 as that was when desensitization was offered at our institution. Their overall treatment courses ranged approximately from 2008 to 2025 with most continuing platinum-based therapy, in combination with taxanes, bevacizumab, PARP inhibitors, or immunotherapy. The cisplatin substitution group spanned from 2004 to 2025 with patients receiving cisplatin, often in combination with doxorubicin, gemcitabine, paclitaxel or bevacizumab. The alternative therapy cohort showed the widest range of treatment durations (1999–2025) with more variability in subsequent treatments, as guided by toxicity, disease progression, or institutional constraints. These patients transitioned to a broad spectrum of therapies, including taxanes, anthracyclines, PARP inhibitors, hormonal therapies, or immunotherapies. Notably, the treatment intervals of these three groups overlapped with patients receiving various similar treatments following initial carboplatin hypersensitivity.

### 3.3 Survival

**Fig. 1:**
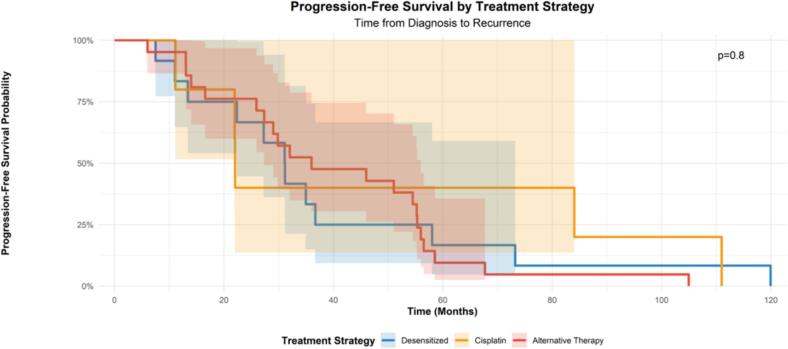
Progression-free Survival.

We first looked at differences in progression free survival amongst the three groupings. Progression-free survival was noted to be significantly longer in desensitized patients at 31.1 months as compared to cisplatin patients at 22.0 months (p = 0.045) (Table 3**,** Fig. 1). Progression-free survival was noted to be longer in alternative therapy patients at 36 months as compared to desensitized patients though was not statistically significant (p = 0.60). Using Cox-proportional hazard modeling, we adjusted for age, BRCA mutation status, and recurrent disease, observing a HR of 0.22 (95 %CI 0.05,0.97), p = 0.045 for use of cisplatin, compared to desensitized patients as the reference group, with reduction in HR for BRCA mutation of 0.40 (95 %CI 0.15, 1.06), p = 0.066 (Table 4).

**Table 3:** Combined Survival Comparisons.

**Comparison**	**Group 1 (n)**	**Median PFS (months)**	**Group 2 (n)**	**Median PFS (months)**	**p-value**
Desensitized vs. Cisplatin	Desensitized (12)	31.1	Cisplatin (5)	22.0	**0.045**
Desensitized vs. Alternate	Desensitized (12)	31.1	Alternate (21)	36.0	0.60
Platinum-Based vs. Alternate	Platinum-Based (17)	31.1	Alternate (21)	36.0	0.70

**Table 4:** Multivariate Cox Proportional Hazards Model.

**Characteristic**	**HR**	**95 % CI**	**p-value**
**Treatment Strategy (Three-Group Model)**			
Desensitized (reference)	—	—	—
Cisplatin	0.22	0.05–0.97	**0.045**
Alternative Therapy	0.73	0.25–2.10	0.6
Age (per SD increase)	0.6	0.34–1.08	0.089
BRCA Mutation	0.4	0.15–1.06	0.066
Recurrent Disease	0.89	0.23–3.42	0.9
Ovarian Cancer (vs Other)	0.66	0.22–2.01	0.5
**Treatment Strategy (Combined Model)**			
Alternative Therapy (reference)	—	—	—
Platinum-based	0.8	0.29–2.15	0.7
Age (per SD increase)	0.62	0.34–1.14	0.12
BRCA Mutation	0.56	0.24–1.32	0.2
Recurrent Disease	0.75	0.20–2.90	0.7
Ovarian Cancer (vs Other)	0.51	0.17–1.51	0.2

Next, we looked at differences in progression free survival when grouped as “platinum-based therapy” versus “alternative therapy” as the reference group. The platinum-based therapy group combined patients who received desensitization and cisplatin into one analysis group. We did not observe significant differences in progression free survival, with median survival of 31.1 months versus 36 months. Using Cox-proportional hazard modeling, we adjusted for age, BRCA mutation status, and recurrent disease, observing a HR of 0.80 (95 % CI 0.29, 2.15), p = 0.7 when comparing “platinum-based therapy” to “alternative therapy” as the reference group.

## 4 Discussion

In this retrospective cohort study, gynecologic cancer patients who experienced a hypersensitivity reaction to carboplatin and were desensitized to carboplatin were noted to have longer progression-free survival as compared to patients who received cisplatin instead. There was no significant difference observed between carboplatin desensitized patients and patients who were transitioned to an alternative therapy, or between patients receiving platinum therapy and alternative therapy when carboplatin and cisplatin patients were combined. To our knowledge, this is the first study that directly compares progression-free survival among gynecologic cancer patients with carboplatin hypersensitivity treated with carboplatin desensitization, cisplatin substitution, or alternative therapies. Our study further reiterates the safety and effectiveness of platinum desensitization therapy for treatment continuation in patients with carboplatin hypersensitivity.

The finding of greater progression-free survival in patients with carboplatin desensitization as compared to cisplatin is a notable one. No prior studies have directly compared these two strategies after a carboplatin reaction, but prior studies have shown that carboplatin and cisplatin have similar efficacy in ovarian cancer, with cisplatin potentially having a slight advantage though with higher toxicity (Lorusso et al., 2014, Ho et al., 2016, Go and Adjei, 1999, Lokich and Anderson, 1998). The progression-free survival advantage noted in carboplatin desensitized patients may be related to patients being able to complete more cycles of therapy due to better tolerability (Altwerger et al., 2018).

The similar progression-free survival outcomes noted between desensitized and alternative therapy patients indicates that desensitization maintains the efficacy of platinum-based therapy without affecting survival outcomes. One reason could be that the alternative therapy patients may have more favorable tumor characteristics for non-platinum-based therapy and thus may be likely to have a better prognosis (Gaillard et al., 2025, Ma et al., 2019, Wilson et al., 2024, Lu and Endometrial, 2020). Similar progression-free survival was further noted between platinum-based therapy and alternative therapy patients—this challenges the paradigm of platinum superiority in the context of carboplatin-hypersensitive patients. These findings persist when controlling for age, BRCA mutation status, and recurrent disease. The fact that this treatment effect is seen even when accounting for BRCA mutation status is notable, as BRCA-mutated gynecologic tumors are generally more sensitive to platinum-based chemotherapy (Dann et al., 2012, Gorodnova et al., 2015, Pennington et al., 2014, Yang et al., 2011).

This study further delineated the clinical phenotypes of patients noted to have carboplatin hypersensitivity. In accordance with prior study findings, a majority of our cohort was observed to have recurrent disease at the time of hypersensitivity (70.2 %), a carboplatin-free interval of 12 months or more (57.4 %), history of adverse reactions to other drugs (76.6 %), advanced stage disease (78.8 %), and serous histology (70.2 %). Prior studies have noted the risk of hypersensitivity to be highest after more than 8 cycles (Tai et al., 2017, Paksoy et al., 2022); our findings align with this report as our median cycle number at time of initial hypersensitivity reaction was 8 cycles. BRCA mutations were noted in 34.0 % of cases—this aligns with findings that BRCA mutations are associated with an increased risk of developing carboplatin hypersensitivity reactions. The prevalence of BRCA1/2 mutations was enriched in our cohort as compared to the prevalence of BRCA1/2 mutations among all women with ovarian cancer (15 %) (US Preventive Services Task Force et al., 2019).

Limitations of our progression-free survival analysis include that the alternative therapies that platinum-based treatment was compared to included patients who received many different therapies such as single-agent paclitaxel, bevacizumab, PARP inhibitors, and radiation. This heterogeneous group of alternative therapies may make it difficult to draw conclusions regarding the survival advantages of platinum-based therapy. Our cohort also includes patients who experienced carboplatin reactions over the past 15 years; during this period of time, treatment guidelines have evolved and new therapies have become available which may confound the disease specific outcomes reported. Additionally, carboplatin desensitization was only offered at our cohort starting 2022 which might create an inherent selection bias among the desensitization group as compared to the cisplatin and alternative treatment groups. Still, patients across the three cohorts were treated over similar time periods, strengthening their comparability.

Another limitation is the relatively small sample size with limited racial/ethnic diversity, as our cohort was composed primarily of white women—this likely limits the generalizability of our findings. We are also limited in our current assessment of somatic and germline mutations. While we have classified the cancer mutations that were known in the patients according to what is most likely (somatic or germline), it is possible that some individual patients were misclassified. Additionally, our overall survival data were not mature, and thus not included in the analysis, which might limit our understanding of our patients’ ultimate oncologic outcomes.

Our study also has several strengths. It is the first study to directly compare oncologic outcomes between patients with carboplatin hypersensitivity reactions who took various treatment paths. Another strength is that our study includes patients with multiple gynecologic malignancies who experienced hypersensitivity reactions to carboplatin, allowing for a comprehensive look at this patient population. Future studies in larger and more diverse patient populations with access to the same treatment options are needed to confirm our findings. Additional studies that incorporate an assessment of toxicity profiles and quality of life between carboplatin desensitization and cisplatin substitution are also necessary (Altwerger et al., 2018, Bergamini et al., 2017, Callahan et al., 2007, Ottaiano et al., 2003). While we did not have the sample size to support an examination of ovarian cancer, endometrial cancer, and cervical cancer separately, studies that are able to assess each malignancy separately may help clarify the trends observed.

In this retrospective single center study, we observed a longer progression-free survival in patients desensitized to carboplatin after a carboplatin hypersensitivity reaction compared to switching to cisplatin, with similar progression-free survival in patients who are desensitized to carboplatin versus switched to alternative therapies. This finding needs to be studied in a larger population of patients. Gynecologic oncologists can integrate these findings into their decision-making and counseling with regards to treatment continuation in patients with carboplatin hypersensitivity. Our study further reiterates the effectiveness of carboplatin desensitization for safe, well-tolerated continuation of carboplatin treatment and delineates the clinical phenotype of carboplatin reactors, with notable characteristics including recurrent disease, carboplatin-free interval of 12 + months, BRCA mutation, and history of adverse reactions to other drugs.

## 5 Conclusions

We conducted the first study on gynecologic malignancy patients with carboplatin hypersensitivity that directly compares progression-free survival between desensitized, cisplatin, and alternative therapy patients. Desensitized patients demonstrated increased progression-free survival as compared to cisplatin patients. This progression-free survival advantage may be due to the increased toxicity profiles noted for cisplatin and comparatively better tolerability of carboplatin desensitization. Progression-free survival was similar between carboplatin-desensitized and alternative therapy patients, as well as platinum-based and alternative therapy patients.

Informed consent was waived as a part of IRB submission—it was not possible to obtain as several of the patients in this retrospective review have passed.

## Funding

This research was supported by grant funding from Chic Awearness and the Vanderbilt Ingram Cancer Center.

## CRediT authorship contribution statement

**Nikita Bastin:** Writing – review & editing, Writing – original draft, Visualization, Validation, Resources, Project administration, Methodology, Investigation, Formal analysis, Data curation, Conceptualization. **Halle Petrie:** Writing – review & editing, Methodology, Investigation, Data curation, Conceptualization. **Marc Robinson:** Writing – review & editing, Data curation. **Amir Javid:** Formal analysis. **Robin Lane:** Methodology, Data curation. **Taryn Boucher:** Writing – review & editing, Data curation, Conceptualization. **Alaina J. Brown:** Writing – review & editing, Project administration, Methodology, Investigation, Data curation, Conceptualization. **Lauren S. Prescott:** Writing – review & editing, Methodology, Investigation, Data curation, Conceptualization. **Elizabeth Phillips:** Writing – review & editing, Methodology, Investigation, Data curation, Conceptualization. **Ronald D. Alvarez:** Writing – review & editing, Writing – original draft, Methodology, Investigation, Data curation, Conceptualization. **Marta Crispens:** Writing – review & editing, Methodology, Investigation, Data curation, Conceptualization. **Cosby A. Stone:** .

## Declaration of competing interest

The authors declare that they have no known competing financial interests or personal relationships that could have appeared to influence the work reported in this paper.
